# Supplementary material for: Molecular and Pathological Profiling of Corresponding Treatment-Naïve and Neoadjuvant Pazopanib-Treated High-Risk Soft Tissue Sarcoma Samples of the GISG-04/NOPASS Study
Source: Biology (Basel). 2021 Jul 9;10(7):639. doi: 10.3390/biology10070639 (PMC8301157; doi:10.3390/biology10070639)
Supplement: Supplementary file 1 [file biology-10-00639-s001.zip › SupplFigS1-TCGA-Sarc-2017.pdf]

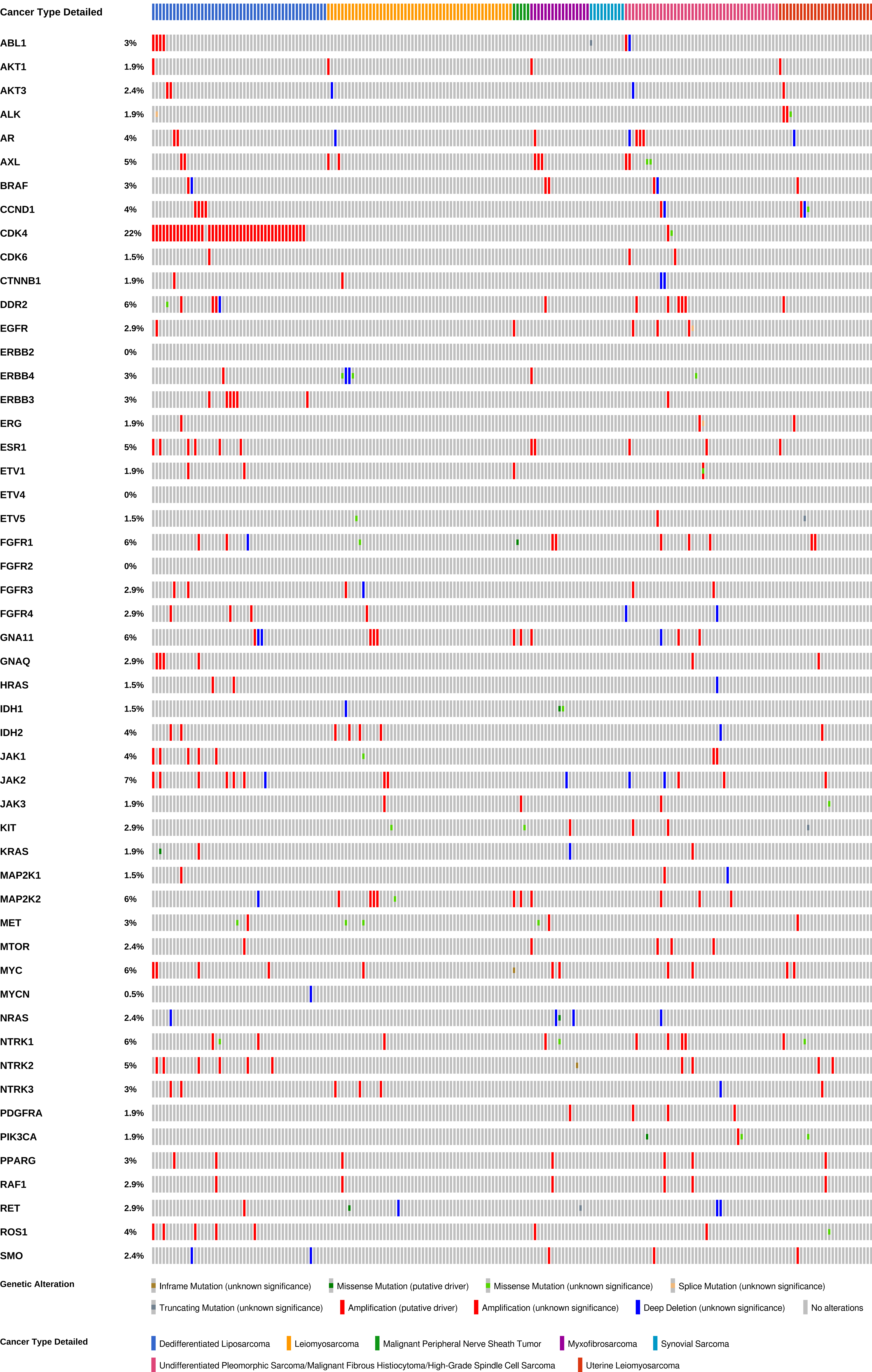
Cancer Type Detailed
ABL1 3%
AKT1 1.9%
AKT3 2.4%
ALK 1.9%
AR 4%
AXL 5%
BRAF 3%
CCND1 4%
CDK4 22%
CDK6 1.5%
CTNNB1 1.9%
DDR2 6%
EGFR 2.9%
ERBB2 0%
ERBB4 3%
ERBB3 3%
ERG 1.9%
ESR1 5%
ETV1 1.9%
ETV4 0%
ETV5 1.5%
FGFR1 6%
FGFR2 0%
FGFR3 2.9%
FGFR4 2.9%
GNA11 6%
GNAQ 2.9%
HRAS 1.5%
IDH1 1.5%
IDH2 4%
JAK1 4%
JAK2 7%
JAK3 1.9%
KIT 2.9%
KRAS 1.9%
MAP2K1 1.5%
MAP2K2 6%
MET 3%
MTOR 2.4%
MYC 6%
MYCN 0.5%
NRAS 2.4%
NTRK1 6%
NTRK2 5%
NTRK3 3%
PDGFRA 1.9%
PIK3CA 1.9%
PPARG 3%
RAF1 2.9%
RET 2.9%
ROS1 4%
SMO 2.4%
Genetic Alteration
Inframe Mutation (unknown significance)
Missense Mutation (putative driver)
Missense Mutation (unknown significance)
Splice Mutation (unknown significance)
Truncating Mutation (unknown significance)
Amplification (putative driver)
Amplification (unknown significance)
Deep Deletion (unknown significance)
No alterations
Cancer Type Detailed
Dedifferentiated Liposarcoma
Leiomyosarcoma
Malignant Peripheral Nerve Sheath Tumor
Myxofibrosarcoma
Synovial Sarcoma
Undifferentiated Pleomorphic Sarcoma/Malignant Fibrous Histiocytoma/High-Grade Spindle Cell Sarcoma
Uterine Leiomyosarcoma
