# Supplementary figures and images for: Molecular and Pathological Profiling of Corresponding Treatment-Naïve and Neoadjuvant Pazopanib-Treated High-Risk Soft Tissue Sarcoma Samples of the GISG-04/NOPASS Study
Source: Biology (Basel). 2021 Jul 9;10(7):639. doi: 10.3390/biology10070639 (PMC8301157; doi:10.3390/biology10070639)

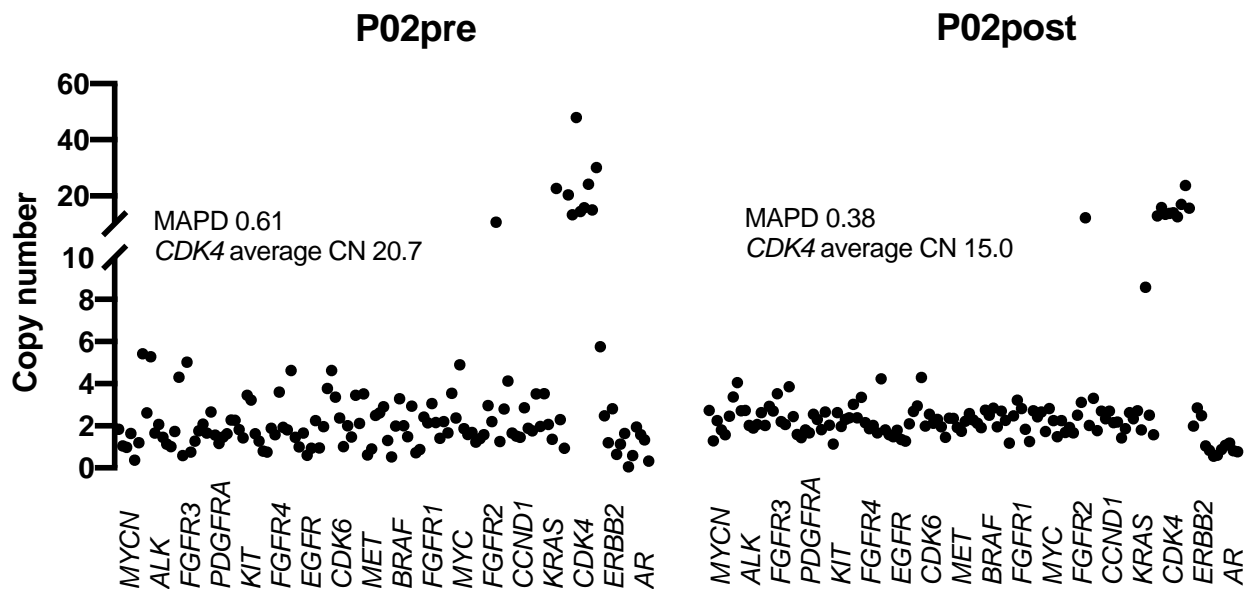

Supplementary Figure 2

Supplement: Supplementary file 1 [file biology-10-00639-s001.zip › SupplFig2_P02-CNplotsCDK4.pdf]
